# Balancing Treatment Efficacy and Cardiovascular Risk: Implications for Psychosocial Care in Men With Castration-Resistant Prostate Cancer

**DOI:** 10.1002/pon.70300

**Published:** 2025-10

**Authors:** Ewan K. Cobran, Umar Afzal, Lanyu Mi

**Affiliations:** Department of Quantitative Health Science, Mayo Clinic College of Medicine and Sciences, Scottsdale, Arizona, USA

## 1 ∣ Introduction

Men with castration-resistant prostate cancer (CRPC) often face a challenging journey characterized not only by disease progression but also by increasingly complex treatment decisions. With the expansion of oral androgen receptor pathway inhibitors (ARPi) as a preferred therapeutic option, questions surrounding their cardiovascular safety and broader impact on quality of life have emerged. Our recent analysis using SEER-Medicare data offers new insights into thromboembolic risks associated with ARPi compared to chemotherapy, insights that may have critical implications for the psychosocial and decision-making landscape in this patient population [1].

## 2 ∣ Understanding the Clinical Context

Treatment for CRPC has evolved rapidly over the past decade, shifting from primarily cytotoxic chemotherapy to the use of more tolerable oral agents such as enzalutamide and abiraterone, given in combination with ADT [2]. These therapies offer not only extended survival but also improved patient-reported outcomes, contributing to the perception that ARPi are inherently safer or easier for patients to tolerate. However, cardiovascular toxicity, particularly thromboembolic events (TE), has emerged as a growing concern in real-world practice, especially in older adults with multiple comorbidities.

Our study of 2779 CRPC patients treated between 2012 and 2016 explored this safety signal by stratifying patients according to pre-existing cardiovascular disease (CVD) and comparing TE risks between ARPi and chemotherapy. Although unadjusted models suggested a lower risk of TE among ARPi users, this difference disappeared after robust adjustment using inverse probability treatment weighting. Importantly, this null finding held true regardless of prior CVD status or previous thromboembolic history.

## 3 ∣ Implications for Psycho-Oncology

At first glance, the absence of increased TE risk with ARPi might appear reassuring. However, the psychosocial implications of these findings extend far beyond the clinical endpoints. Patients and caregivers navigating CRPC treatment are often burdened with decisional uncertainty, particularly when safety trade-offs are poorly understood. Cardiovascular events, even when rare, can dramatically impact quality of life, induce anxiety, and result in hospitalization, all of which can erode the emotional well-being of both patients and families [3].

Furthermore, the perception of ARPi as a gentler alternative to chemotherapy may lead patients to underestimate their risks. As psycho-oncology professionals, we must recognize how this misalignment between perception and reality can create tension in the shared decision-making process. Patients may feel blindsided by cardiovascular complications or experience decisional regret if adverse events occur after choosing a supposedly low-risk therapy [4].

## 4 ∣ Cognitive Burden and Health Literacy

Older adults with CRPC often have concurrent cognitive challenges, limited health literacy, or diminished capacity to evaluate nuanced treatment risks [5]. Discussions around cardiovascular risk, especially when framed in probabilistic terms, can be difficult for patients to understand and interpret. Clinicians may unintentionally simplify risk profiles, reinforcing misconceptions that oral therapies are universally safer. Psycho-oncology clinicians can play a critical role here by advocating for better communication strategies, encouraging open discussions about side effect profiles, and supporting informed decision-making aligned with patients’ values.

## 5 ∣ Equity Considerations and Vulnerable Populations

Another dimension warranting attention is health equity. The SEER-Medicare cohort includes a racially and socioeconomically diverse population, and it is well-documented that Black men with prostate cancer are more likely to experience worse cardiovascular outcomes and face barriers to high-quality care [6]. As newer therapies are adopted, disparities in both access and cardiovascular risk mitigation may become further entrenched. Our findings underscore the importance of integrating psychosocial support services and cardio-oncology consultation into standard care, particularly for medically complex and historically marginalized populations.

## 6 ∣ Conclusions and Future Directions

Our study adds a nuanced perspective to the cardiovascular safety discussion surrounding ARPi. While we found no increased adjusted risk of thromboembolic events compared to chemotherapy, the implications for patients’ emotional well-being, treatment perceptions, and decision-making are profound. As ARPi use continues to rise, psycho-oncology professionals must engage proactively to help patients interpret safety data, navigate risk-benefit discussions, and address the emotional toll of complex cancer care decisions.

Future work should explore how cardiovascular risk perceptions influence treatment choices, how clinicians communicate risk, and how psychosocial interventions can support informed, value-concordant care in men with advanced prostate cancer. The integration of cardiovascular and psychosocial care is not only a clinical imperative, but also central to truly patient-centered oncology.
